# Anti-*Helicobacter pylori*, Antioxidant, Antidiabetic, and Anti-Alzheimer’s Activities of Laurel Leaf Extract Treated by Moist Heat and Molecular Docking of Its Flavonoid Constituent, Naringenin, against Acetylcholinesterase and Butyrylcholinesterase

**DOI:** 10.3390/life13071512

**Published:** 2023-07-05

**Authors:** Aisha M. H. Al-Rajhi, Husam Qanash, Majed N. Almashjary, Mohannad S. Hazzazi, Hashim R. Felemban, Tarek M. Abdelghany

**Affiliations:** 1 Department of Biology, College of Science, Princess Nourah bint Abdulrahman University, P.O. Box 84428, Riyadh 11671, Saudi Arabia; amoalrajhi@pnu.edu.sa; 2 Department of Medical Laboratory Science, College of Applied Medical Sciences, University of Ha’il, Hail 55476, Saudi Arabia; h.qanash@uoh.edu.sa; 3 Department of Medical Laboratory Sciences, Faculty of Applied Medical Sciences, King Abdulaziz University, Jeddah 22254, Saudi Arabia; malmashjary@kau.edu.sa (M.N.A.); mshazzazi@kau.edu.sa (M.S.H.); hrfelemban@kau.edu.sa (H.R.F.); 4 Hematology Research Unit, King Fahd Medical Research Center, King Abdulaziz University, Jeddah 22254, Saudi Arabia; 5 Special Infectious Agents Unit-BSL3, King Fahd Medical Research Center, King Abdulaziz University, Jeddah 21362, Saudi Arabia; 6 Botany and Microbiology Department, Faculty of Science, Al-Azhar University, Cairo 11725, Egypt

**Keywords:** *Laurus nobilis* L., anti-*Helicobacter pylori*, antioxidant, antidiabetic, anti-Alzheimer, naringenin

## Abstract

It is worth noting that laurel (*Laurus nobilis* L.) contains several pharmacologically and nutritionally active compounds that may differ according to the pretreatment process. The current study is designed to clarify the effect of moist heat on the phenolic and flavonoid constituents and anti-*Helicobacter pylori*, antioxidant, antidiabetic, and anti-Alzheimer’s activities of laurel leaf extract (LLE). Unmoist-heated (UMH) and moist-heated (MH) LLEs showed the presence of numerous flavonoid and phenolic constituents, although at different levels of concentration. MH significantly induced (*p* < 0.05) the occurrence of most compounds at high concentrations of 5655.89 µg/mL, 3967.65 µg/mL, 224.80 µg/mL, 887.83 µg/mL, 2979.14 µg/mL, 203.02 µg/mL, 284.65 µg/mL, 1893.66 µg/mL, and 187.88 µg/mL, unlike the detection at low concentrations of 3461.19 µg/mL, 196.96 µg/mL, 664.12 µg/mL, 2835.09 µg/mL, 153.26 µg/mL, 254.43 µg/mL, 1605.00 µg/mL, 4486.02 µg/mL, and 195.60 µg/mL using UMH, for naringenin, methyl gallate, caffeic acid, rutin, ellagic acid, coumaric acid, vanillin, ferulic acid, and hesperetin, respectively. Chlorogenic acid, syringic acid, and daidzein were detected in the UMH LLE but not in the MH LLE, unlike pyrocatechol. The anti-*H. pylori* activity of the UMH LLE was lower (23.67 ± 0.58 mm of inhibition zone) than that of the MH LLE (26.00 ± 0.0 mm of inhibition zone). Moreover, the values of MIC and MBC associated with the MH LLE were very low compared to those of the UMH LLE. Via MBC/MIC index calculation, the UMH and MH LLEs showed cidal activity. The MH LLE exhibited higher anti-biofilm activity (93.73%) compared to the anti-biofilm activity (87.75%) of the MH LLE against *H. pylori*. The urease inhibition percentage was more affected in the UMH LLE compared to the MH LLE, with significant (*p* < 0.05) IC_50_ values of 34.17 µg/mL and 91.11 µg/mL, respectively. Promising antioxidant activity was documented with a very low value of IC_50_ (3.45 µg/mL) for the MH LLE compared to the IC_50_ value of 4.69 µg/mL for the UMH LLE and the IC_50_ value of 4.43 µg/mL for ascorbic acid. The MH LLE showed significantly higher (*p* < 0.05) inhibition of α-glucosidase and butyrylcholinesterase activities, with IC_50_ values of 9.9 µg/mL and 17.3 µg/mL, respectively, compared to those of the UMH LLE at 18.36 µg/mL and 28.92 µg/mL. The molecular docking of naringenin showed good docking scores against acetylcholinesterase 1E66 and butyrylcholinesterase 6EMI, indicating that naringenin is an intriguing candidate for additional research as a possible medication for Alzheimer’s disease.

## 1. Introduction

There is still debate regarding the biological activities of many natural extracts and how to use them in the right way, as well as the methods of extracting them to release large quantities of active substances. In the current investigation, the *Laurus nobilis* plant was used due to the importance of this plant in human life. *L. nobilis*, belonging to the lauraceae family, is a plant commonly dispersed in the Mediterranean Region and some other countries, such as Algeria, Turkey, Greece, Portugal, Morocco, Spain, Mexico, Italy, Greece, Morocco, U.S.A., and Belgium [1].

This plant, also known as sweet bay, bay, Roman laurel, bay laurel, and daphne, is aromatic and evergreen; is utilized as a cooking spice in several countries, including Western and Asian countries; and is applied in traditional medicine as a stimulant, an antiseptic, a stomachic digestive, and a sudorific asset. Some studies reported the biological activities of *L. nobilis* extracts as well as *L. nobilis* essential oils (EOs); for instance, Sakran et al. [2] revealed the antimicrobial activities of an ethanol extract of *L. nobilis* against *Escherichia coli*, *Salmonellae typhi*, and *Staphylococcus aureus*, with different levels of inhibition zones and minimum inhibitory concentrations (MICs). The antifungal and antioxidant activities of *L. nobilis* flower EOs were documented [3,4].

*L. nobilis* leaf EOs were applied for food preservation to prevent microbial contamination, and da Silveira et al. [5] evaluated the effect of *Yersinia enterocolitica* and *E. coli* on the quality of fresh Tuscan sausage stored at 7 °C for 14 days and supplemented with *L. nobilis* leaf EOs. Moreover, Marques et al. [6] preserved seafood, meat, and some agricultural products via the addition of *L. nobilis* leaf EOs because of their antimicrobial and antioxidant properties. The antagonistic potential of *L. nobilis* leaf extract was documented against *Proteus vulgaris* and *Staphylococcus saprophyticus* in fresh lamb meat [7]. Therefore, lamb meat’s shelf life increased from 1 to 3 days at room temperature and from 6 to 13 days at refrigeration temperature as a result of spraying fresh lamb meat with 10% (*v*/*v*) *L. nobilis* leaf extract.

Other previous studies reported that *L. nobilis* extract was utilized as an additive in cosmetic and food products due to the existence of aromatic and flavor constituents [8,9]. Pharmacological effects such as immune-modulating and cytotoxic effects were also attributed to *L. nobilis* extract [3]. The activity of *L. nobilis* extract may depend on several factors, such as the extracted organ of the plant and the used solvent. Fidan et al. [10] reported that *L. nobilis* leaf EO exhibited antimicrobial activity against nearly all tested bacteria as well as fungi, while only *S. aureus* from the tested microorganisms was inhibited when using twig EOs.

In the present study, the impact of moist heat on the phenolic and flavonoid constituents as well as some of the biological activities of *L. nobilis* leaf extract was investigated. The effects of other processes on *L. nobilis* extract had been studied; for example, drying via air and a microwave was effective regarding the chemical contents and antimicrobial activity of *L. nobilis* EOs [11]. Similarly, Khodja et al. [12] studied the effects of two kinds of drying approaches, microwave-assisted drying ranging from 180 to 900 W and drying via air and an oven at temperatures ranging from 40 °C to 120 °C, on the total phenolic content and antioxidant activity of *L. nobilis* leaf extract. Extraction methods, such as hydro-distillation, hydro-steam distillation, ohmic-assisted hydro-distillation, and microwave-assisted hydro-distillation, had been experimented with and evaluated to study their effects on the yield, antioxidant activity, and chemical composition of *L. nobilis* EOs [13], which indicated that microwave-assisted hydro-distillation gave higher quantities of oxygenated monoterpenes.

*Helicobacter pylori* is one of the most common chronic bacterial pathogens of human beings at all age stages. Numerous illnesses of the upper gastrointestinal tract, such as gastric inflammation and gastric and duodenal ulcers, have been associated with *H pylori* [14,15]. Gastritis development and peptic ulceration are caused by ammonia release from *H. pylori* urease that acts as virulence and colonization factors. However, while the antimicrobial activity of *L. nobilis* leaf extract has been studied extensively on several microorganisms, there is limited research on *H. pylori*. As mentioned previously, *H. pylori* was inhibited by *L. nobilis* leaf extract with an inhibition zone of 24 mm at 200 mg/mL [16]. Secondary metabolites of the plant have been recommended as possible alternatives for the treatment of *H*. *pylori* as well as other microbes [17,18,19,20,21,22]. Alzheimer’s disease (AD) is a chronic neurodegenerative disease that is considered one of the most common health issues in the world, and its prevalence is increasing among the elderly population [23]. Investigators have continued to develop and discover bioactive substances of a natural origin in the creation of novel drugs for efficient treatment as a result of the continuous rise in the incidence of neurological disorders in humanity. AD is associated with acetylcholine depletion in the brain, which is due to acetylcholinesterase (AChE) and butyrylcholinesterase (BChE) catalytic activities [24], thereby making AChE and BChE potential targets for drug design in the management of AD. Most published reports have explained the biological activities of different fresh or dried organs of *L. nobilis* extract, but without considering the effect of moist heat on plant constituents. There is scarce information about the impact of moist heat on the biological activities of *L. nobilis* extract. Therefore, in this investigation, we present the effects of moist heat on the chemical constituents of *L.nobilis*, and its anti-*H. pylori*, antioxidant, antidiabetic, and anti-Alzheimer’s activities

## 2. Materials and Methods

### 2.1. Chemical Used

Acetonitrile (molar mass: 41.053 g·mol^−1^, density: 0.786 g/cm^3^ at 25 °C, solubility in water: miscible, and purity: 99.5%), dimethylsulfoxide (DMSO) (molar mass: 78.13 g·/mol, density: 1.1004 g/cm, solubility in water: miscible, and purity: 99%), 2,2-diphenyl-1-picrylhydrazyl (DPPH) (molar mass: 394.32 g/mol, density: 1.4 g/cm^3^, solubility in water: insoluble, and purity: 99.13%), methanol (molar mass: 32.04 g/mol, density: 0.792 g/cm^3^, solubility in water: miscible, and purity: 99.85%), and Mueller–Hinton agar were purchased from Sigma-Aldrich (Steinheim, Germany).

### 2.2. Sample Collection and Effect of Moist Heat

Air-dried leaves of laurel (*Laurus nobilis* L.) were collected from a supermarket in Saudi Arabia. The identification of the plant was achieved by the taxonomist Prof. Marei A. Hamed from the Botany and Microbiology Department, Faculty of Science, Al-Azhar University, Egypt. The laurel leaves were washed with distilled water to remove any dust particles and then re-dried in air. The re-dried air leaves were divided into two parts: the first part was kept at it was for 15 min at room temperature and then used for unmoist- heat (UMH) treatment, while the second part, at the same time, was autoclaved at 80 °C for 15 min and used for the moist heat (MH) treatment. This was followed by the extraction of each part using methanol. The UMH and MH laurel leaf extracts (LLEs) (200 g for each) were grinded and then mixed with 500 mL of methanol with a magnetic stirrer for 1 day. The mixture was centrifuged for 10 min at 5000 rpm. The obtained supernatant was concentrated utilizing a rotary evaporator, completely concentrated to obtain 5 g of crude extract, re-dissolved in DMSO, and then kept at 4 °C for further investigations.

### 2.3. HPLC Analysis of Phenolic and Flavonoid Contents of UMH and MH LLEs

Phenolic and flavonoid compound identification of the UMH and MH LLEs was carried out utilizing an Agilent Series 1260 device (Agilent, Santa Clara, CA, USA) for High-Performance Liquid Chromatography (HPLC). The HPLC device consisted of a quaternary HP pump (series 1200), an auto-sampling injector, a solvent degasser, the 1100 ChemStation software (DFAR 252.227-7014), an Eclipse C18 column (4.6 mm, 250 mm i.d., 5 μm, and 40 °C) and an ultraviolet (UV) detector adjusted to 280 nm for phenolic acids and to 330 nm for flavonoids, correspondingly. Separation of flavonoid was performed by utilizing 50 mM of H_3_PO_4_ (pH of 2.5) (solution A) and acetonitrile/acetic acid (40/60, *v*/*v*) (solution B) as the mobile phases in the gradient, in the following subsequent stages: isocratic elution 95%/5% (A/B) for 0–5 min; in the gradient linear from 95%/5% (A/B) to 50%/50% (A/B) for 5–55 min; isocratic elution 50%/50% (A/B) for 55–65 min; and in the gradient linear from 50%/50% (A/B) to 95%/5% (A/B) for 65–67 min, with 0.7 mL/min of the mobile phase as the flow rate. The following solvent system was utilized for the separation of phenolic acids, consisting of 2.5% aqueous acetic acid (A), 8% aqueous acetic acid (B), and acetonitrile (C) in the following subsequent stages: 5% B at 0 min, 10% B at 20 min, 30% B at 50 min, 50% B at 55 min, 50% B and 50% C at 60 min, 100% B at 100 min, and 100% C from 110 min to 120 min. The solvent flow rate was 1 mL/min of solvent. A total of 5 µL of the separated compounds was injected into the HPLC device. Quantitative detection of the separated compounds was calculated according to the data of standard compounds. The solution of standard stock of flavonoid and phenolic compounds was prepared at different dilutions (10–80 µg/mL) in methanol solvent, followed by injection into the HPLC device [25].

### 2.4. Total Phenolic and Flavonoid Content Detection

The UMH or MH LLE was added to Folin–Ciocalteau’s reagent (10% *v*/*v*), and then Na_2_CO_3_ (7.5%) was added to the reaction mixture, followed by incubation for 40 min at 45 °C. At 765 nm, the absorbance was measured to determine the total phenol content, which was expressed as mg of gallic acid equivalent (GAE)/g of dry weight of the extract. On the other hand, the total flavonoid content was determined via the addition of the extract to 0.5 mL of methanol, 500 μL of 10% aluminum chloride, 50 μL of 1 M potassium acetate, and 1.4 mL of distilled water. This was followed by incubation for 30 min at 25 °C. At 415 nm, absorbance was measured to determine the total flavonoid content, which was expressed as mg of quercetin equivalent (QE)/g of dry weight of the extract.

### 2.5. Anti-H. pylori Activity of UMH and MH Extracts of L. nobilis Leaves

The agar-well diffusion method using Mueller–Hinton (MH) medium was used to test the UMH and MH LLEs against *H. pylori* based on standard 2 of McFarland’s turbidity. An inoculum of *H. pylori* was seeded onto the surface of the M-H-containing agar using a sterile cotton swab. The agar wells were then drilled using a sterile 6 mm drill and filled with 100 μL of each extract at 100 mg/mL. Some wells were filled with 10% dimethylsulfoxide (DMSO) as a negative control and others with 0.05 μg/mL clarithromycin as a standard control. All plates were refrigerated at 4 °C for 30 min in order to allow the samples to spread before the growth of *H. pylori* began. The plates containing the inoculums were incubated in a GasPak™ anaerobic system “Oxoid” under suitable conditions, including a microaerobic environment at 37 °C, and an incubation time of 72 h. The zones of inhibition that appeared were then measured in millimeters [14].

### 2.6. Assay of Minimal Inhibitory Concentration (MIC) and Minimal Bactericidal Concentration (MBC)

Mueller–Hinton broth was used as the micro-dilution broth to determine the MICs of the UMH and MH LLEs against *H. pylori*. Various serial dilutions of the UMH and MH LLEs were prepared, ranging from 0.98 to 1000 μg/mL. A 96-well polystyrene microtiter plate was used, and 200 μL of each dilution of the UMH and MH LLEs was dispensed per well. Fresh *H. pylori* inoculum was prepared in sterile NaCl (0.85%) to achieve the required McFarland turbidity of 1.0. A total of 2 μL of tested organisms was inoculated into each well to give a final dose of 5 × 10^4^ colony-forming units/mL. The plates were incubated at 37 °C for 72 h. The MIC was then visually assessed to determine how effectively the overall growth of the tested microorganisms was inhibited. Each micro-plate containing an inoculum of *H. pylori* without the UMH and MH LLEs was utilized as a positive control, while the micro-plates containing the UMH or MH LLE without *H. pylori* were used as a negative control [26]. The MBC was detected from the micro-dilution plates utilized for the MIC test. Aliquots (10 μL) of each well without visible growth were transferred to Mueller–Hinton agar plates and incubated at 37 °C for 72 h. The lowest concentration of the UMH and MH LLEs that fully inhibited *H. pylori* growth on the plates is known as the MBC. To characterize whether the UMH and MH LLEs had bacteriostatic or bactericidal abilities, the MBC/MIC ratio was recorded. If the MBC/MIC ratio of any extract is no more than four times the value of the MIC, the extract is called a bactericidal agent [27].

### 2.7. Anti-Biofilm Activity of UMH and MH LLEs

In 96-well polystyrene flatbottom plates, the impact of the UMH and MH LLEs on the development of *H. pylori* biofilms was assessed. In brief, 300 μL of freshly inoculated trypticase soy yeast broth (TSY) with a final concentration of 10^6^ CFU/mL was aliquoted into each well of a microplate and cultivated in the presence of previously calculated sub-lethal doses of MBC (75, 50, and 25%). As the controls, wells with the medium and those with methanol and no extracts were employed. For 48 h, the plates were incubated at 37 °C. After incubation, the supernatant was removed, and free-floating *H. pylori* cells were completely cleaned from each well using sterile distilled H_2_O. The plates were then allowed to air dry for 30 min, and the biofilm that had developed was stained for 15 min at room temperature using a 0.1% crystal violet aqueous solution. Following incubation, the extra stain was washed away with sterile distilled H_2_O. Finally, the attached dye to *H. pylori* cells was solubilized via the addition of 250 μL of ethanol (95%) to each well, and then absorbance was measured using a microplate reader at a wavelength of 570 nm after 15 min of incubation. The biofilm inhibition % was recorded using the following formula:

$$\mathrm{Biofilm}\;\mathrm{inhibition}\;\%\;=1-\left(\frac{\mathrm{Absorbance}\;\mathrm{of}\;\mathrm{extract}-\mathrm{absorb}.\;\mathrm{Blank}}{\mathrm{Absorbance}\;\mathrm{of}\;\mathrm{control}-\mathrm{bsorb}.\;\mathrm{Blank}}\right)\times 100$$

Absorbance of the media only represents the blank sample, absorbance of *H. pylori* from the treatment represents the extract, and absorbance of *H. pylori* without any treatment represents the control [28].

### 2.8. Urease Inhibition by UMH or MH LLE

The reaction mixture solution was composed of 850 μL of urea, the UMH or MH LLE (up to 1000 µg/mL), and 100 mM of phosphate buffer (pH of 7.4) to reach a total value of 985 μL. The reaction mixture started via the addition of urease enzyme (15 μL) and was then measured by determining the concentration of ammonia at 60 min, utilizing 500 μL of solution A (composed of 0.5 g of phenol and 2.5 mg of sodium nitroprusside dissolved in 50 mL of distilled H_2_O) and 500 μL of solution B (composed of 250 mg of NaCl and 820 μL of 5% NaOCl hypochlorite dissolved in 50 mL of H_2_O) for 30 min at 37 °C. Uninhibited urease activity was taken as 100% of the control activity. According to the Berthelot spectrophotometric method, absorbance was measured at 625 nm, and then the urease inhibition percentage was recorded using the following formula:

$$\mathrm{Urease}\;\mathrm{inhibition}\;\%\;=1-\left(\frac{\mathrm{Absorbance}\;\mathrm{of}\;\mathrm{extract}\;\mathrm{or}\;\mathrm{positive}\;\mathrm{control}}{\mathrm{Absorbance}\;\mathrm{of}\;\mathrm{enzyme}\;\mathrm{as}\;\mathrm{control}}\right)\times 100$$

The concentration that stimulates an inhibition value halfway between the minimum and maximum responses of the extract (IC_50_) was detected by checking the inhibition influence of different concentrations of the extract in the test. Urease inhibition was recorded using hydroxyurea as the standard compound [29].

### 2.9. DPPH Radical Scavenging Activity for Antioxidant Activity Assessment of UMH and MH LLEs

The capacity of the LLEs to scavenge free radicals was evaluated using the 1,1-diphenyl-2-picrylhydrazyl (DPPH) assay. Briefly, 50 μL of the extract solution in water (1.95 to 1000 µg/mL) was combined with 2950 μL of a 60 M solution of DPPH in methanol. After giving the mixture a good shake, it was left to stand at room temperature for 30 min. Then, using a Varian 50 Bio spectrophotometer, absorbance was determined at 517 nm. A higher free radical scavenging capacity of the reaction mixture was indicated by a lower absorbance value. A standard antioxidant (ascorbic acid) was applied in this experiment. The following equation was used to determine the ability of the extracts to scavenge DPPH radicals:

$$\mathrm{DPPH}\;\mathrm{scavenging}\;\left(\%\right)=\frac{C0-\;\mathrm{LE}\;1}{C0\;}\times 100$$

where C_0_ means the absorbance of the control reaction, while LE1 means the absorbance of the LLEs. The concentration of the LLEs required to inhibit 50% (IC_50_) of DPPH free radicals was recorded using a log dose–inhibition curve [30].

### 2.10. Assay of α-Glucosidase Inhibition by UMH and MH LLEs

The LLEs were evaluated for α-glucosidase inhibitory potential according to the approach presented by Pistia-Brueggeman and Hollingsworth [31] with minor modifications. The reaction mixture containing 50 μL of each extract at different concentrations, ranging from 1.97 to 1000 μg/mL, was mixed with the α-glucosidase (10 μL of 1 U/mL) enzyme solution. Phosphate buffer (125 μL of 0.1 M at a pH of 6.8) was added to the reaction mixture, and the mixture was then incubated at 37 °C for 20 min. At the end of the incubation period, 20 μL of 1 M *p*-nitrophenyl-*α*-D-glucopyranoside (pNPG) as a substrate was added to start the reaction, followed by incubation for 30 min. Then, 50 μL of 0.1 N Na_2_CO_3_ was added to terminate the reaction. Absorbance at 405 nm was measured via a Biosystm 310 plus spectrophotometer. The α-glucosidase inhibition % was calculated using the following formula:

$$\alpha -\mathrm{Glucosidase}\;\mathrm{inhibition}\;\%\;=\left(\frac{\mathrm{OD}\;\mathrm{BLANK}-\mathrm{OD}\;\mathrm{extract}}{\mathrm{OD}\;\mathrm{BLANK})}\right)\times 100$$

One unit of enzyme can be expressed as the quantity of α-glucosidase required for the formation of p-Nitrophenol (one μmol) from p-NPG per min. This concentration is requisite to prevent 50% of the enzyme activity (IC_50_) and is calculated utilizing a regression equation obtained through plotting the concentration (1.97–1000 μg/mL) and inhibition (%) for different concentrations.

### 2.11. Butyrylcholinesterase Inhibition Assay

Butyrylcholinesterase (BTchI) inhibition was assayed according to the Ellman method with some modifications [32,33]. The buffer and solutions for BChE were freshly made. This involved preparing 0.022 M S-butyrylthiocholine iodide (BTchI) solution (7.0 mg of BTchI was dissolved in 1 mL of water) and 0.44 U/mL of BChE solution (2.9762 mg of BChE enzyme was dissolved in 6.746 mL of buffer at a pH of 8.0). To achieve a final concentration of 1000 μg/mL, each LLE was first dissolved in DMSO and then in distilled water to obtain a concentration of 44 mg/mL. The BChE inhibition assay was determined via measuring absorbance using a microplate reader; 200 μL of the buffer, 5 μL of BChE enzyme, 5 μL of Ellman’s reagent 5,5′-dithiobis-2-nitrobenzoic acid (DTNB), and 5 μL of the LLE at a concentration of 40 mg/mL were combined and kept in a solution for 15 min at 30 degrees Celsius in a temperature-controlled water bath. The enzymatic reaction was then started by adding 5 μL of the BTchI substrate solution to the mixture. Absorbances were measured at 410 nm utilizing the microplate reader at 45 s intervals 13 times at a controlled 30 °C. Using the following formula, the measured absorbance was used to determine the enzymatic inhibition:

$$\mathrm{Butyrylcholinesterase}\;\mathrm{inhibition}\;\;\left(\%\right)=100-\left[\left(\frac{R\;\mathrm{extract}}{R\;\max}\right)\right]\times 100$$

where R extract means the change rate in the absorbance of the test containing the LLE (Δabs/Δtime), while R max means the maximum change rate in the absorbance of the blank sample without any inhibitor.

### 2.12. Molecular Docking Investigation

The crystal structures of acetylcholinesterase (PDB code 1E66) and butyrylcholinesterase (PDB code 6EMI) proteins were downloaded from the protein data bank (https://www.rcsb.org/, accessed on 30 March 2021). Naringenin was docked via the MOE 2019 software. Both receptor and compound creation and optimization were carried out using the software’s default method for structural optimization. Hydrogen atoms were added after the elimination of all water molecules around the proteins. Then, naringenin optimized its shape with the lowest binding energy while using the MMFF94x force field to achieve this optimization. Alpha-site spheres were created using the site finder in the MOE module. The five best binding configurations with flexible molecular rotation were created using the DFT-optimized structure of naringenin. The free binding energy (S, in kcal/mol), which represents the binding affinity, was ranked using hydrogen bonds that developed between the proteins and naringenin. Validation was performed by re-docking naringenin into the binding pocket and measuring the root-mean-square deviation (RMSD) between positions.

### 2.13. Statistical Analysis

The outcomes are presented as mean ± SD (standard deviation), and the results were taken as the average of three replicates. The statistical analysis was performed using the computer programs Microsoft Excel version 365 and SPSS v.25 (Statistical Package for the Social Sciences version 25.00). Quantitative data with parametric distribution between the different treatments were analyzed utilizing one-way analysis of variance (ANOVA) and Tukey’s post hoc test, at 0.05 probability level.

## 3. Results and Discussion

### 3.1. Phenolic and Flavonoid Characterization of LLEs

**Figure 1:**
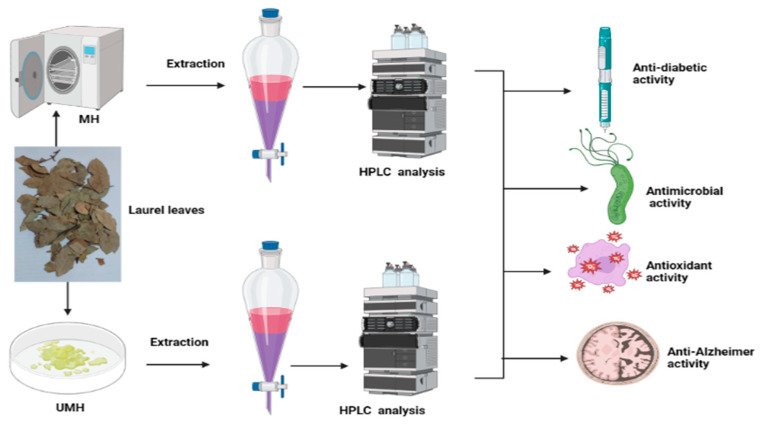
Planned experiments for moist-heated (MH) and unmoist-heated laurel leaves.

**Figure 2:**
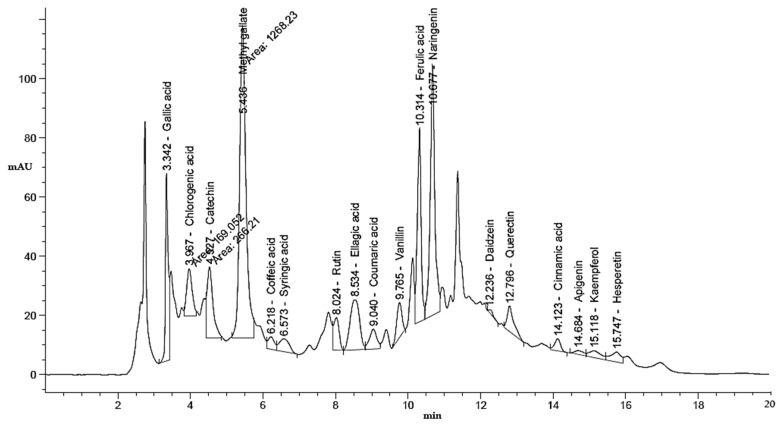
HPLC chromatogram of identified phenolic and flavonoid constituents in unmoist-heated laurel leaf extract.

**Figure 3:**
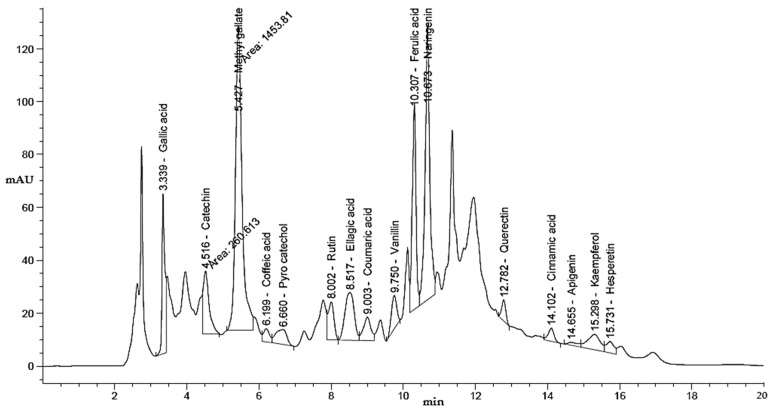
HPLC chromatogram of identified phenolic and flavonoid constituents in moist-heated laurel leaf extract.

This study examined the influence of moist heat on the phenolic and flavonoid contents of LLE compared to air-dried LLE, as well as anti-*H. pylori*, antioxidant, antidiabetic, and anti-Alzheimer’s activities (Figure 1). The LLE, either unmoist-heated (UMH) or moist-heated (MH), was enriched with several flavonoid and phenolic compounds (Table 1 and Figure 2 and Figure 3). Naringenin, methyl gallate, catechin, and ellagic acid were the most detected compounds with the highest concentrations, while apigenin and cinnamic acid were detected with the lowest concentrations in either the UMH or MH LLE. Surprisingly, significantly high (*p* < 0.05) concentrations of 3967.65 µg/mL, 224.80 µg/mL, 887.83 µg/mL, 2979.14 µg/mL, 203.02 µg/mL, 284.65 µg/mL, 1893.66 µg/mL, 5655.89 µg/mL, 51.22 µg/mL, 95.03 µg/mL, 547.19 µg/mL, and 187.88 µg/mL were detected for methyl gallate, caffeic acid, rutin, ellagic acid, coumaric acid, vanillin, ferulic acid, naringenin, cinnamic acid, apigenin, kaempferol, and hesperetin in the MH LLE, compared to their concentrations of 3461.19 µg/mL, 196.96 µg/mL, 664.12 µg/mL, 2835.09 µg/mL, 153.26 µg/mL, 254.43 µg/mL, 1605.00 µg/mL, 4486.02 µg/mL, 43.25 µg/mL, 88.55 µg/mL, 206.95 µg/mL, and 195.60 µg/mL, respectively, in the UMH LLE. These results indicated that moist heat promoted the release of phenolic and flavonoid compounds. Juániz et al. [34] reported that heat treatments of vegetables increased the concentration of phenolic constituents and proposed that thermal damage of cell walls and sub-cellular compartments throughout the cooking process stimulates the discharge of constituents. However, chlorogenic acid was detected at a high concentration of 1157.51 µg/mL, besides syringic acid and daidzein, in the UMH LLE, but it was not detected in the MH LLE. These disappeared compounds might be unstable under heat treatment or might have transformed into other compounds. Therefore, pyrocatechol was detected at a concentration of 868.77 µg/mL only in the MH LLE but not in the UMH LLE. Table 1 shows the total contents of phenolic compounds and flavonoids in the UMH and MH LLEs. The present findings showed that the UMH LLE contained less total phenolic content (1.87 ± 0.33 mg GAE/g) and total flavonoid content (0.68 ± 0.10 mg QE/g) than the MH LLE (2.65 ± 0.17 mg GAE/g and 1.05 ± 0.10 mg QE/g, respectively).

Previously, Routray and Orsat [35] mentioned that exposure of natural extracts to higher microwave power resulted in the degradation of some phenolic constituents. Dobroslavić et al. [36] found the presence of variable amounts of 29 phenolic and flavonoid compounds in LLE, with the most abundance being quercetin glycosides and kaempferol. Moreover, they noticed that temperature increases from 40 to 80 °C resulted in higher total phenolic content. Higher total phenolic content was obtained as a result of exposure of *L. nobilis* to boiling water for 3 h, compared to the amount obtained at room temperature after 72 h [37]. In another report, Bulut Kocabas et al. [38] noticed that the total phenolic content of *L. nobilis* leaves exposed to heated water at 80 °C for 45 min was 10-fold more than that obtained at room temperature for 24 h, with apigenin and its glycosides representing the most detected flavones in the LLE [39]. But in our study, apigenin was detected at a low concentration. Four phenolic compounds, including gallic acid, coumaric acid, pyrogallol, and resorcinol, were detected in the *L. nobilis* extract via HPLC [40]. Alejo-Armijo et al. [39] mentioned that various phenolic compounds, such as flavonoids, tannins (proanthocyanidins), phenolic acids, and lignans, were associated with LLE, particularly flavonoids, which represent the most abundant constituents with a variety of identified compounds. Fidan et al. [10] mentioned that the differences in chemical constituents in *L. nobilis* are perhaps due to the dissimilar genotypes, the planted and climatic conditions where the plants were cultivated, and the extraction process [10].

### 3.2. Anti-H. pylori Activity

**Figure 4:**
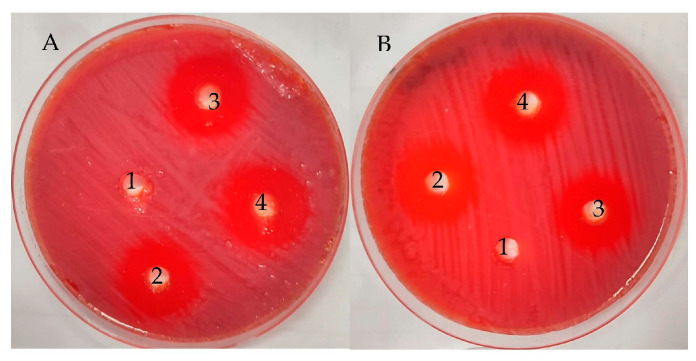
Antimicrobial activity of UMH (**A**) and MH (**B**) laurel leaf extracts (1, negative control; 2, positive control; 3 and 4, two wells of the plant extract).

**Figure 5:**
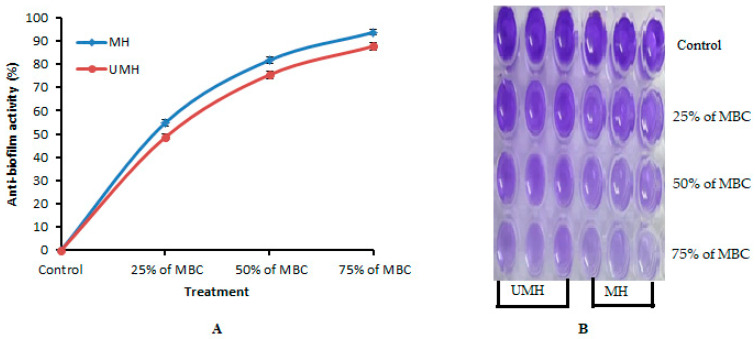
Anti-biofilm activity of MU LLE and UMH LLE against *H. pylori*: (**A**) microtiter plate reveals changes in stain color as a pointer of icreased anti-biofilm formation of *H. Pylori* (**B**) under different treatments of media + *H. Pylori* (Cont.); 25% of MBC, 50% of MBC, and 75% of MBC.

**Figure 6:**
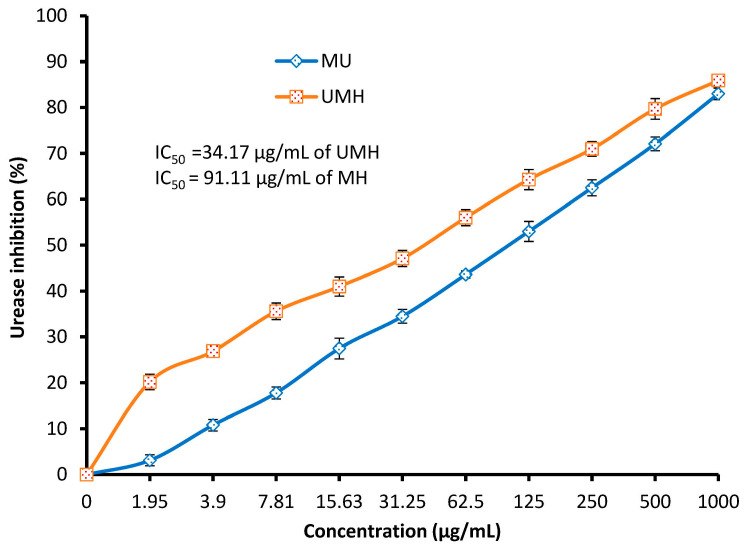
Urease inhibition at different concentrations of MU LLE and UMH LLE.

The UMH LLE exhibited anti-*H. pylori* activity but less than the MH LLE, with inhibition zones of 23.67 ± 0.58 mm and 26.00 ± 0.0 mm, respectively (Table 2 and Figure 4). The inhibitory potential of both UMH and MH LLEs was good compared to the positive control that showed an inhibition zone of 20.33 ± 0.58 mm against *H. pylori*. Our results were consistent with previous findings [16], where acetone and hexane extracts of *L. nobilis* inhibited *H. pylori* with inhibition zones of 24 mm and 26 mm, respectively. Dobroslavić et al. [41] demonstrated the antimicrobial activity of LLE. The presence of numerous flavonoid and phenolic compounds in the LLE is one of the main reasons for the anti-*H. pylori* activity. For instance, ellagic acid [42], naringenin [43], and ferulic acid [15] exhibited anti-*H. pylori* activity. Several investigations have shown that flavonoids play a critical role in inhibiting the spread of *H. pylori* [15,44,45]. The MH LLE showed greater inhibitory activity than the MH LLE, which might be related to the fact that the most analyzed compounds via HPLC were detected at the highest concentration compared to the fresh extract. It is very clear that the MIC and MBC values of the MH LLE were significantly (*p* < 0.05) lower (1.9 µg/mL) than the MIC and MBC values of 7.8 µg/mL of the UMH LLE (Table 2). Via the calculation of the MBC/MIC index, it is obvious that both UMH and MH LLEs possess cidal activity due to the MBC/MIC index being one (Table 2). The biofilm formation of *H. pylori* was more affected by the MH LLE than the UMH LLE. The anti-biofilm activity increment with an increase in MBC concentrations of the MH and UMH LLEs. In the same line, it was observed that the anti-biofilm activity of the MH LLE was higher than the UMH LLE; for instance, the anti-biofilm activity was 93.73 and 87.75% at 75% for the MH LLE’s MBC and the UMH LLE’s MBC, respectively (Figure 5A). The anti-biofilm activity of the MH and UMH LLEs was also indicated by the change in the color of stained *H. pylori* biofilm in the microtiter plate, which was dependent on anti-biofilm activity (Figure 6B).

### 3.3. Urease Inhibition

In the stomach, *H. pylori* uses urease enzyme to neutralize and protect itself from acidic conditions via ammonia production, and the activity of urease is necessary for *H. pylori* colonization. Urease inhibition represents one mechanism that determines the anti-*H. pylori* activity of antimicrobial compounds. In the present study, urease inhibition was documented using both the MH and UMH LLEs with different inhibition percentages depending on the concentration of the extracts. Unlike the results of anti-*H. pylori* activity, MIC, MBC, and anti-biofilm activity, which were more affected by the MH LLE, urease inhibition was more affected by the UMH LLE compared to the MH LLE (Figure 6). For instance, at 1.95, 62.5, 250, and 500 µg/mL, urease inhibition was 20.2, 56.0, 71.0, and 85.9% in the UMH LLE, while it was 3.1, 43.6, 62.5, and 83.0%, respectively, in the MH LLE. A lower significant (*p* < 0.05) value of IC_50_ (34.17 µg/mL) was recorded in the UMH LLE compared to the IC_50_ (91.11 µg/mL) of the MH LLE. These results can be explained by the possibility of the presence of another mechanism of anti-*H. pylori* activity, such as inhibition of DNA gyrase, N-acetyltransferase, and dihydrofolate reductase associated with the presence of compounds in the extract, or that the mechanism of the compounds in the UMH LLE may differ from the mechanism of the compounds in the MH LLE. Hydroxyurea was applied as a standard compound for urease inhibition, which showed an IC_50_ of 37.5 µg/mL. Surprisingly, the obtained IC_50_ value of the MH LLE was less (34.17 µg/mL) than that of hydroxyurea. This outcome suggests the capability of this extract to be applied as a promising extract to manage illnesses of the gastrointestinal tract alone or in combination with authorized drugs for anti-*H. pylori* treatment. As mentioned in the results of the HPLC analysis, naringenin was the main detected compound in both the MH and UMH LLEs, and this compound at a concentration of 300 µg/mL showed urease inhibition (34%) of *H. pylori* [44]. According to Biglar et al. [46], *L. nobilis* extract was tested against *H. pylori* growth and exhibited an attractive value of IC_50_ (48.69 μg/mL) for urease inhibition.

### 3.4. Antioxidant Activity

The presence of many flavonoid and phenolic compounds in the LLEs made us expect antioxidant activity, but not to the extent noted in Table 3, suggesting that the flavonoid and phenolic constituents present in the LLEs have a high level of hydroxylation, which is manifested in their high ability to provide protons and, therefore, stabilize DPPH. The antioxidant activity measured via DPPH scavenging % increased with an increase in extract concentration up to 1000 µg/mL of the UMH and MH LLEs, giving values of 95.5% and 97.1%, respectively (Table 3). Papageorgiou et al. [47] reported that the extracted phenolic acids of LLE reflected antioxidant activity. The present results showed that the antioxidant capacity of the MH LLE was higher than that of the UMH LLE, but with a slight difference. Not surprisingly, the IC_50_ (3.45 µg/mL) of the MH LLE was less than the IC_50_ value (4.69 µg/mL) of the UMH LLE, but the most surprising thing is that it is less than the IC_50_ value (4.43 µg/mL) of ascorbic acid. LLE, because of its wide presence of bioactive molecules, is an excellent source of antioxidant potential for pharmaceutical and cosmetic applications [41]. Our investigation on the antioxidant activity of LLE was consistent with many studies using UMH LLE [36,40,48], but there were no reported studies about MH LLE. The efficacy of conventional heat-reflux extraction (CRE), ultrasound-assisted extraction (UAE), and microwave-assisted extraction (MAE) was studied to evaluate the antioxidant activity of LLE, and the highest antioxidant capacity was obtained via CRE [41].

### 3.5. Antidiabetic Activity

**Figure 7:**
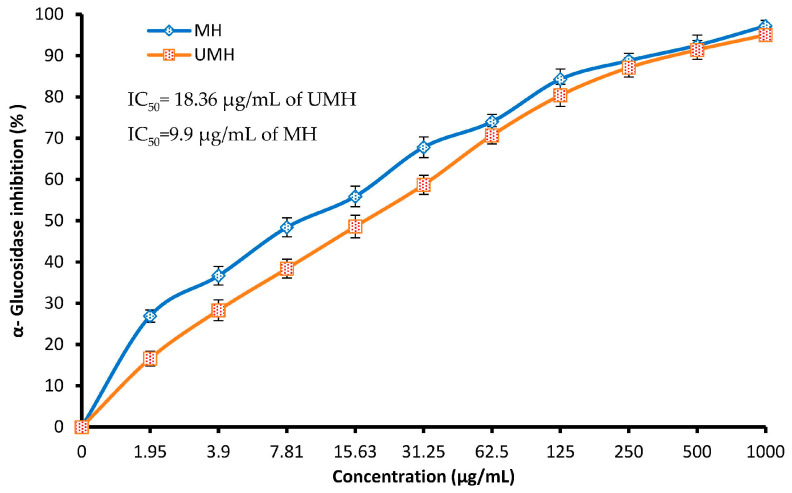
Antidiabetic activity at different concentrations of MU LLE and UMH LLE.

α-glucosidase activity inhibition is considered a successful strategy for controlling the level of blood sugar and regulating food-related hyperglycemia. In our investigation, both the MH and UMH LLEs were effective inhibitors of glucosidase in a dose-dependent manner (Figure 7). However, the MH LLE showed the highest inhibitory activity toward this enzyme compared to the UMH LLE, particularly at low concentrations of up to 31.25 µg/mL; for instance, at 1.95, 7.81, and 31.25 µg/mL using the MH LLE, glucosidase inhibition was 26.9, 48.4, and 55.9%, while using the UMH LLE, α-glucosidase inhibition was 16.6, 38.4, and 58.7%, respectively. The values of IC_50_ of the two extracts were promising, but from the present findings, the IC_50_ of the MH LLE was significantly (*p* < 0.05) more promising (9.9 µg/mL) compared to the IC_50_ of the UMH LLE (18.36 µg/mL). These results may be because of the presence of some compounds in the UMH LLE. According to a study performed by Khan et al. [49], the lipid and glucose profiles of diabetic patients were improved because of treatment with powdered *L. nobilis* leaves in capsule form. In another study, *L. nobilis* (bay laurel) extracted with different solvents inhibited α-glucosidase metabolic activity [50]. Our finding endorses the utilization of LLE, particularly MH, for further investigations to identify their potential for regulating type II diabetes. Earlier investigations have shown that several natural plants contain rich constituents with valuable inhibitory potential toward α-glucosidase. According to a previous study [51], quercetin and rutin could inhibit glucosidase via binding with alpha-glucosidase to form a new complex. A recent report showed that catechin and quercetin displayed significant inhibitory potential toward α-glucosidase with different IC_50_ values of 12.78 and 92.87 μg/mL, respectively, while some compounds such as gallic acid and ellagic acid displayed moderate α-glucosidase inhibitory capacity, with IC_50_ values of 102.35 and 222.80 μg/mL, respectively [52].

### 3.6. Anti-Alzheimer’s Activity

**Figure 8:**
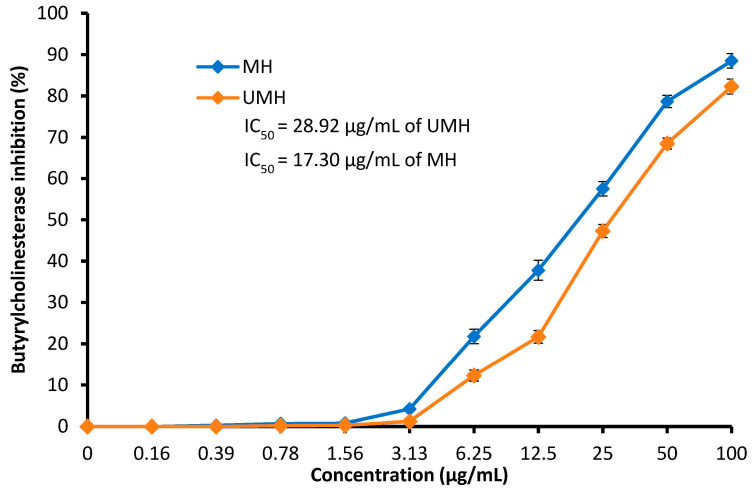
Anti-Alzheimer’s activity at different concentrations of MU LLE and UMH LLE.

In the current study, inhibition of butyrylcholinesterase (BChE) was recorded as a result of exposure to the LLEs (Figure 8). This enzyme is used as a marker of Alzheimer’s disease (AD) development. Recently, Falade et al. [53] reported that *L. nobilis* may be considered a promising agent for the nutraceutical management of AD. Figure 8 shows that the inhibition of BChE activity increases with an increase in both the UMH and MH LLEs up to 100 µg/mL, with inhibition of 82.3 and 88.5%, respectively. The MH LLE has the best inhibitory potential for BChE, with an IC_50_ of 17.3 µg/mL, compared to the UMH LLE, with an IC_50_ of 28.92 µg/mL, at all concentrations used; these results may be related to the high concentrations of the most detected compounds in the MH LLE as recorded in the HPLC analysis. Inhibition of two enzymes, namely BChE and acetylcholinesterase (AChE), was observed (100% inhibition) as a result of exposure to 30 µg/mL of *L. nobilis* extract, with IC_50_ values of 4.76 ± 0.36 µg/mL and 4.21 ± 0.50 μg/mL, respectively [53]. In vivo, phytoconstituents of *L. nobilis* had inhibitory action against BChE and AChE in scopolamine-induced rats [54].

### 3.7. Molecular Docking of Naringenin with Acetylcholinesterase and Butyrylcholinesterase

**Figure 9:**
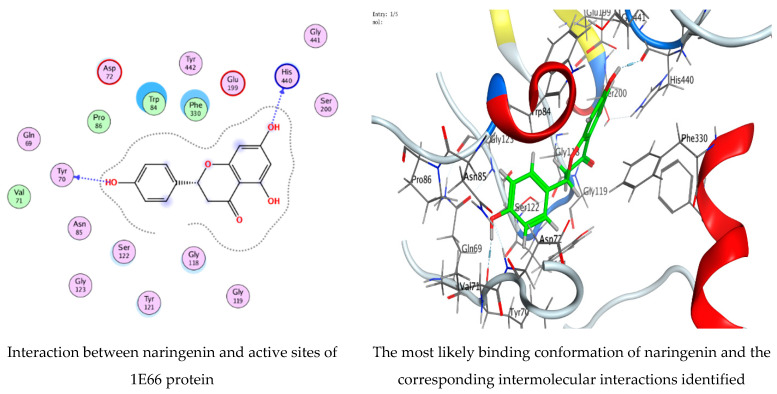

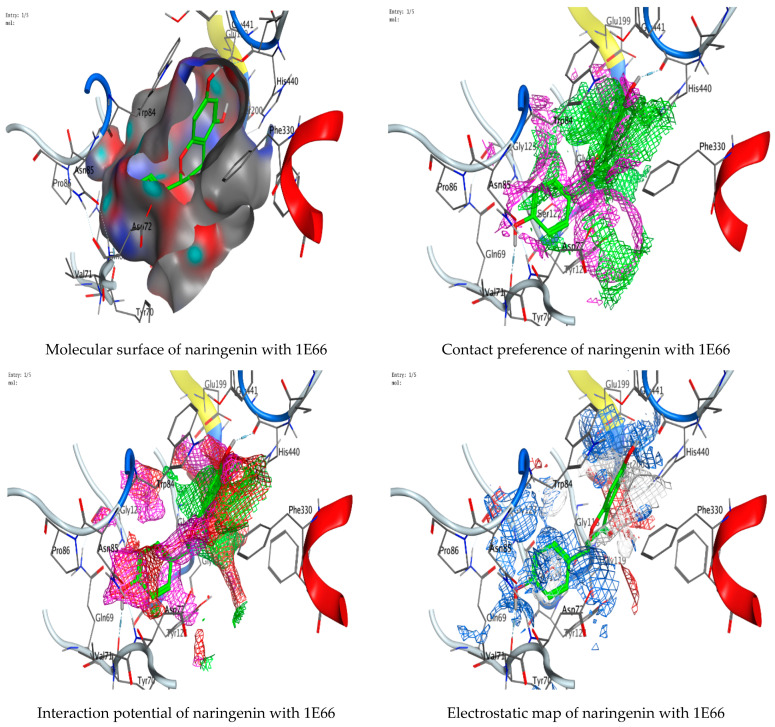
Molecular docking process of naringenin with 1E66.

**Figure 10:**
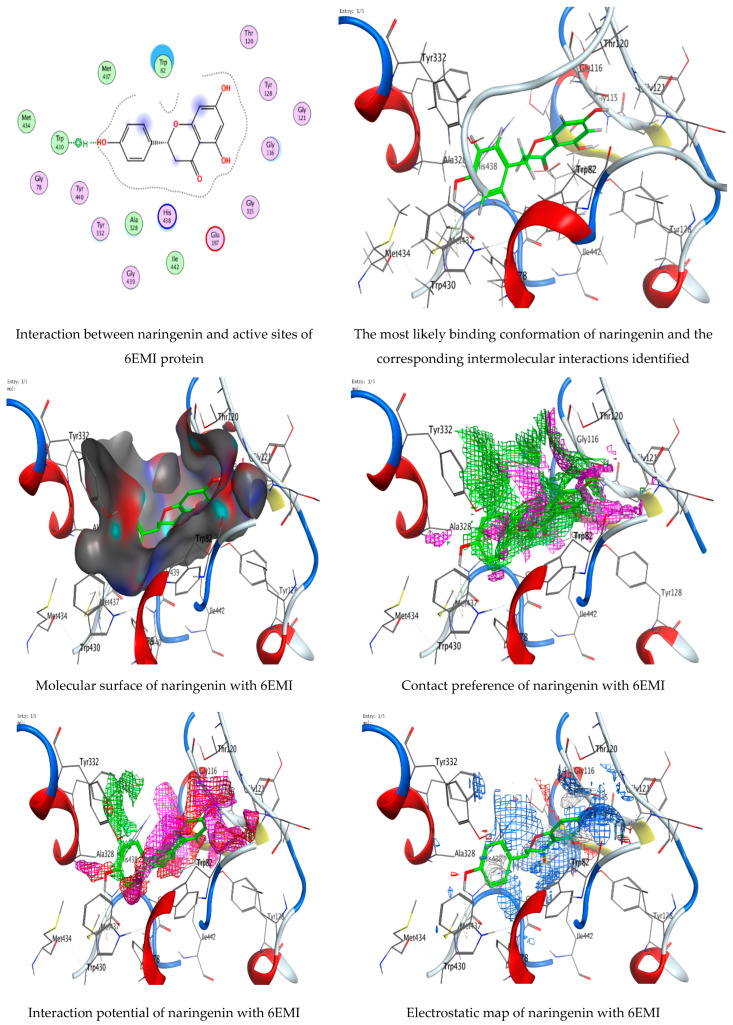
Molecular docking process of naringenin with 6EMI.

The technology of molecular docking is an effective approach to imagine the interaction among ligands of micromolecular size and receptors of macromolecular size, allowing the detection of probable binding sites and the ligands’ steric conformations. Thus, in the current investigation, a molecular docking technique was employed to predict a potent ligand (naringenin) that could inhibit acetylcholinesterase 1E66 and butyrylcholinesterase 6EMI. The investigation also aimed to gain a better understanding of how the inhibitor interacts with the protein binding sites. The investigation was conducted using naringenin, and the RMSD values (Figure 9 and Figure 10) were found to be 0.978201 Å and 0.733427 Å for docking with acetylcholinesterase 1E66 and butyrylcholinesterase 6EMI, respectively, demonstrating that the MOE-Dock approach is trustworthy for docking this inhibitor and that our docking method is valid for the investigated inhibitor. The binding interactions with (1E66) revealed that the ligand forms strong hydrogen bonding interactions through O 29 and O 30 with TYR 70 and HIS 440 residues at a distance of 2.81 Å and 2.82 Å, respectively. Moreover, it is observed that naringenin binds well with butyrylcholinesterase 6EMI via O 29 through the TRP 430 residue in the active pocket of the protein. An analysis of the binding interactions (Table 4 and Table 5) illustrated the binding of naringenin with 1E66 and 6EMI proteins and the formation of several hydrogen bonds. Naringenin achieves favorable binding to both target proteins with negative scores of −6.78716 Kcal/mole and −6.14549 Kcal/mole. All docking scores and energies are presented in Table 6 and Table 7. Molecular docking interactions were performed in other studies to support the biological activities of several natural constituents [30,55,56]. Recently, several phytoconstituents were docked against AChE and BChE [54].

## 4. Conclusions

To the best of our knowledge, there are no previous reports on the relation between the effects of moist heat and the contents of flavonoid and phenolic compounds, anti-*H. pylori*, antioxidant, antidiabetic, and anti-Alzheimer’s activities of LLE. In the present investigation, moist heat was effective in improving the release and the yield of flavonoid and phenolic compounds, which was accompanied by increases in their biological activities. The molecular docking study led to the discovery of significant ligand interactions with regard to the target protein’s binding site. The main detected compound (naringenin) in the LLEs was docked molecularly with acetylcholinesterase and butyrylcholinesterase as indicators of Alzheimer’s disease development. As a result of our investigation, we concluded that naringenin, which was theoretically examined here, exhibited good docking scores and binding interactions. Therefore, naringenin may be an effective therapeutic candidate, and their effectiveness against acetylcholinesterase (1E66) and butyrylcholinesterase (6EMI) may be boosted. LLE under the effect of moist heat is an excellent base for releasing more valuable compounds with potential applications in the pharmacological and food industries. Although natural extracts have been documented as brilliant substitutes for synthetic drugs, pharmacological utilization is challenging due to the diversity and complication of bioactive constituents present in such extracts. Therefore, the special influences of natural extracts at the level of cell therapy need to be better and more deeply investigated to complement the deficiencies in in vitro investigations in future studies.

## Figures and Tables

**Table 1:** Identified phenolic and flavonoid compounds in moist-heated (MH) and unmoist-heated (UMH) laurel leaf extracts, with the total phenolic and flavonoid contents.

Compound	UMH Laurel Leaf Extract				MH Laurel Leaf Extract			
	Retention Time	Area	Area (%)	Conc. (µg/mL)	Retention Time	Area	Area (%)	Conc. (µg/mL)
Gallic acid	3.34	332.50	7.68	1435.58	3.339	325.41	6.79	1405.00
Chlorogenic acid	3.97	169.05	3.90	1157.51	4.169	0.00	0.00	0.00
Catechin	4.53	266.21	6.15	3296.24	4.516	260.61	5.44	3226.94
Methyl gallate	5.44	1268.23	29.30	3461.19	5.427	1453.81	30.34	3967.65
Caffeic acid	6.22	51.22	1.18	196.96	6.199	58.46	1.22	224.80
Syringic acid	6.57	83.73	1.93	283.90	6.528	0.00	0.00	0.00
Pyrocatechol	6.72	0.00	0.00	0.00	6.660	120.75	2.52	868.77
Rutin	8.02	114.38	2.64	664.12	8.002	152.91	3.19	887.83
Ellagic acid	8.53	305.87	7.06	2835.09	8.517	321.42	6.71	2979.14
Coumaric acid	9.04	97.16	2.24	153.26	9.003	128.70	2.69	203.02
Vanillin	9.77	116.26	2.69	254.43	9.750	130.07	2.71	284.65
Ferulic acid	10.31	469.84	10.85	1605.00	10.307	554.34	11.57	1893.66
Naringenin	10.68	744.04	17.19	4486.02	10.673	938.07	19.58	5655.89
Daidzein	12.24	10.24	0.24	31.64	12.312	0.00	0.00	0.00
Quercetin	12.80	110.03	2.54	757.79	12.782	60.99	1.27	420.02
Cinnamic acid	14.12	46.96	1.08	43.25	14.102	55.61	1.16	51.22
Apigenin	14.68	23.27	0.54	88.55	14.655	24.97	0.52	95.03
Kaempferol	15.12	53.38	1.23	206.95	15.298	141.13	2.95	547.19
Hesperetin	15.75	67.11	1.55	195.60	15.731	64.46	1.35	187.88
Total phenol	1.87 ± 0.33 mg GAE/g				2.65 ± 0.17 mg GAE/g			
Total flavonoid	0.68 ± 0.10 mg QE/g				1.05 ± 0.10 mg QE/g			

**Table 2:** Inhibitory activity, MIC, and MBC of MH and UMH LLEs against *H. pylori*.

Mean Inhibition Zone (mm)				MIC (µg/mL)		MBC (µg/mL)		MBC/MIC Index	
UMH	MH	Control	Negative	UMH	MH	UMH	MH	UMH	MH
23.67 ± 0.58	26.00 ± 0.0	20.33 ± 0.58	0.0 ± 0.0	7.8 ± 0.1	1.9 ± 0.17	7.8 ± 0.35	1.9 ± 0.1	1.0	1.0

**Table 3:** DPPH scavenging % of ascorbic acid, MH LLE, and UMH LLE.

Concentration (µg/mL)	Ascorbic Acid	UMH LLE	MH LLE
	DPPH Scavenging %	DPPH Scavenging %	DPPH Scavenging %
1000	97.0 ±0.004	95.5 ± 0.003	97.1 ± 0.006
500	94.2 ± 0.001	93.3 ± 0.001	94.7 ± 0.001
250	90.0 ± 0.004	87.6 ± 0.005	90.3 ± 0.003
125	83.1 ± 0.004	81.7 ± 0.002	85.2 ± 0.005
62.50	76.4 ± 0.004	75.6 ± 0.003	77.7 ± 0.003
31.25	69.3 ± 0.002	68.9 ± 0.003	71.0 ± 0.006
15.63	62.5 ± 0.003	61.80.003	64.5 ± 0.004
7.81	55.2 ± 0.002	54.6 ± 0.004	57.8 ± 0.004
3.90	48.3 ± 0.002	47.6 ± 0.003	50.5 ± 0.007
1.95	40.2 ± 0.002	39.9 ± 0.004	42.1 ± 0.002
0	0.0 ± 0.000	0.0 ± 0.000	0.0 ± 0.000
IC_50_	4.43 µg/mL	4.69 µg/mL	3.45 µg/mL

**Table 4:** Docking scores and energies of naringenin with the crystal structure of acetylcholinesterase 1E66.

Mol	rseq	mseq	S	rmsd_refine	E_conf	E_place	E_score1	E_refine	E_score2
Naringenin	1	1	−6.78716	0.978201	−39.4713	−76.5186	−13.1741	−36.5393	−6.78716
Naringenin	1	1	−6.67799	1.277979	−34.1638	−68.3821	−12.2916	−36.1206	−6.67799
Naringenin	1	1	−6.62979	1.202798	−40.401	−90.799	−12.9332	−38.2054	−6.62979
Naringenin	1	1	−6.58196	0.838779	−33.5025	−72.8311	−12.7582	−35.0069	−6.58196
Naringenin	1	1	−6.55275	1.554429	−39.5703	−71.615	−12.3801	−32.3347	−6.55275

**Table 5:** Docking scores and energies of naringenin with the crystal structure of butyrylcholinesterase 6EMI.

Mol	rseq	mseq	S	rmsd_refine	E_conf	E_place	E_score1	E_refine	E_score2
Naringenin	1	1	−6.14549	0.733427	−39.739	−86.8222	−11.8804	−26.7900	−6.14549
Naringenin	1	1	−6.02164	1.344088	−38.5364	−65.6819	−11.5795	−26.8067	−6.02164
Naringenin	1	1	−5.88513	0.995142	−39.3925	−65.6398	−11.7741	−26.4780	−5.88513
Naringenin	1	1	−5.81087	1.068273	−37.007	−80.6394	−12.5956	−27.5993	−5.81087
Naringenin	1	1	−5.74699	1.258355	−37.1225	−74.7382	−12.0082	−24.9304	−5.74699

**Table 6:** Interaction of naringenin with the crystal structure of acetylcholinesterase 1E66.

Mol	Ligand	Receptor	Interaction	Distance	E (kcal/mol)
Naringenin	O 29	O TYR 70 (A)	H-donor	2.81	−2.7
	O 31	O HIS 440 (A)	H-donor	2.82	−3.3

**Table 7:** Interaction of naringenin with the crystal structure of butyrylcholinesterase 6EMI.

Mol	Ligand	Receptor	Interaction	Distance	E (kcal/mol)
Naringenin	O 29	5-ring TRP 430 (A)	H-Pi	4.03	−0.6

## Data Availability

Not applicable.
